# Implant Removal in the Management of Prosthetic Joint Infection by *Staphylococcus aureus*: Outcome and Predictors of Failure in a Large Retrospective Multicenter Study

**DOI:** 10.3390/antibiotics10020118

**Published:** 2021-01-26

**Authors:** Joan Gómez-Junyent, Jaime Lora-Tamayo, Josu Baraia-Etxaburu, Mar Sánchez-Somolinos, Jose Antonio Iribarren, Dolors Rodriguez-Pardo, Julia Praena-Segovia, Luisa Sorlí, Alberto Bahamonde, Melchor Riera, Alicia Rico, Mª Dolores del Toro, Laura Morata, Javier Cobo, Luis Falgueras, Natividad Benito, Elena Muñez, Alfredo Jover-Sáenz, Carles Pigrau, Javier Ariza, Oscar Murillo

**Affiliations:** 1 Department of Infectious Diseases, Hospital Universitari de Bellvitge, IDIBELL, Universitat de Barcelona, 08907 L’Hospitalet de Llobregat, Spain; gjunyent@hotmail.com (J.G.-J.); jariza@bellvitgehospital.cat (J.A.); 2 Department of Internal Medicine, Hospital Universitario 12 de Octubre, 28041 Madrid, Spain; sirsilverdelea@yahoo.com; 3 Department of Infectious Diseases, Hospital Universitario de Basurto, 48013 Bilbao, Spain; baraiajosu@gmail.com; 4 Department of Microbiology and Infectious Diseases, Hospital General Universitario Gregorio Marañón, 28009 Madrid, Spain; msanchezs35@yahoo.es; 5 Department of Infectious Diseases, Hospital Universitario Donostia, Universidad del País Vasco (EHU/UPV), 20014 San Sebastián, Spain; joseantonio.iribarrenloyarte@osakidetza.eus; 6 IIS BioDonostia, 20014 San Sebastián, Spain; 7 Infectious Diseases Department, Hospital Universitari Vall d’Hebron, Universitat Autònoma de Barcelona, 08035 Barcelona, Spain; dolorodriguez@vhebron.net (D.R.-P.); cpigrau@vhebron.net (C.P.); 8 Clinical Unit of Infectious Diseases, Microbiology and Preventive Medicine, Hospital Universitario Virgen del Rocío, 41013 Seville, Spain; juliapraena@gmail.com; 9 Department of Infectious Diseases, Hospital del Mar, Institut Hospital del Mar d’Investigacions Mèdiques (IMIM), 08003 Barcelona, Spain; lsorli@parcdesalutmar.cat; 10 Department of Internal Medicine, Hospital El Bierzo, 24411 Ponferrada, Spain; med007783@me.com; 11 Fundació Institut d’Investigació Sanitària Illes Balears, Hospital Universitari Son Espases, 07120 Palma, Spain; melchor.riera@ssib.es; 12 Unit of Infectious Diseases and Clinical Microbiology, Hospital Universitario La Paz, 28046 Madrid, Spain; alicia.rico@salud.madrid.org; 13 Clinical Unit of Infectious Diseases, Microbiology and Preventive Medicine, Hospital Universitario Virgen Macarena, Department of Medicine, Universidad de Sevilla, Instituto de Biomedicina de Sevilla (IBiS), 41009 Seville, Spain; mdeltoro@us.es; 14 Department of Infectious Diseases, Hospital Clínic de Barcelona, Universitat de Barcelona, IDIBAPS, 08036 Barcelona, Spain; lmorata@clinic.cat; 15 Department of Infectious Diseases, Hospital Universitario Ramón y Cajal, 28034 Madrid, Spain; javier.cobo@salud.madrid.org; 16 Department of Infectious Diseases, Hospital Universitari Parc Taulí, 08208 Sabadell, Spain; lfalgueras@tauli.cat; 17 Infectious Diseases Unit, Hospital de la Santa Creu i Sant Pau- Institut d’Investigació Biomèdica Sant Pau, 08025 Barcelona, Spain; nbenito@santpau.cat; 18 Department of Medicine, Universitat Autònoma de Barcelona, 08025 Barcelona, Spain; 19 Unit of Infectious Diseases, Department of Internal Medicine, Hospital Universitario Puerta de Hierro, 28220 Madrid, Spain; elmuru@gmail.com; 20 Territorial Unit of Nosocomial Infections, Hospital Universitari Arnau de Vilanova, 25198 Lleida, Spain; ajover.lleida.ics@gencat.cat; 21 Spanish Network for Research in Infectious Diseases (REIPI RD16/0016/0005), 41071 Sevilla, Spain

**Keywords:** *Staphylococcus aureus*, prosthetic joint infection, implant removal, outcome, rifampin

## Abstract

Objectives: To compare the characteristics and outcomes of cases with acute prosthetic joint infection (PJI; early post-surgical or hematogenous) by *Staphylococcus aureus* managed with implant removal (IRm) or debridement and retention (DAIR). To analyze the outcomes of all cases managed with IRm (initially or after DAIR failure). Methods: Retrospective, multicenter, cohort study of PJI by *S. aureus* (2003–2010). Overall failure included mortality within 60 days since surgery and local failure due to staphylococcal persistence/relapse. Results: 499 cases, 338 initially managed with DAIR, 161 with IRm. Mortality was higher in acute PJI managed initially with IRm compared to DAIR, but not associated with the surgical procedure, after propensity score matching. Underlying conditions, hemiarthroplasty, and methicillin-resistant *S. aureus* were risk factors for mortality. Finally, 249 cases underwent IRm (88 after DAIR failure); overall failure was 15.6%. Local failure (9.3%) was slightly higher in cases with several comorbidities, but independent of previous DAIR, type of IRm, and rifampin treatment. Conclusions: In a large multicenter study of *S. aureus* PJI managed with IRm, failure was low, but mortality significant, especially in cases with acute PJI and underlying conditions, but not associated with the IRm itself. Rifampin efficacy was limited in this setting.

## 1. Introduction

Prosthetic joint infection (PJI) is a serious complication after joint replacement [1]. *Staphylococcus aureus* represents almost a third of all episodes [2], mostly associated with acute PJI (early post-surgical and hematogenous infections) [3], but also with chronic post-surgical infections. 

Surgery is central for the optimal management of PJI by *S. aureus*, with two main strategies: debridement, antibiotics, and implant retention (DAIR), or implant removal (IRm) [3,4]. Observational studies have analyzed the outcome of DAIR [5,6,7,8,9], but the prognosis of IRm, generally performed in chronic PJI or after DAIR failure, has not been extensively evaluated [10,11]. Some authors have suggested that IRm as salvage therapy may lead to poorer outcomes compared with an initial management with IRm [12,13]. The role of rifampin is not formally established, contrasting with its benefits in DAIR [8,14].

Previously, the prognosis of the largest case-series of staphylococcal PJI managed with DAIR was analyzed [8]. However, the characteristics and outcome of cases treated with IRm were not reported in that analysis. 

Therefore, our aim was to revise this large multicenter study with the objectives of (i) analyzing the subcohort of cases with acute PJI to compare the characteristics and outcomes of those initially managed with IRm or DAIR; and (ii) evaluating the outcomes of the subgroup of all cases managed with IRm, initially or as salvage therapy after DAIR failure including the role of rifampin.

## 2. Results

During the study period, 561 cases were initially identified to have PJI by *S. aureus*, but 62 cases had exclusion criteria. Thus, 499 cases were finally included: 325 (65.1%) with early post-surgical (EA) PJI, 75 (15.0%) with hematogenous PJI, and 99 (19.8%) with chronic post-surgical PJI. 

**Figure 1:**
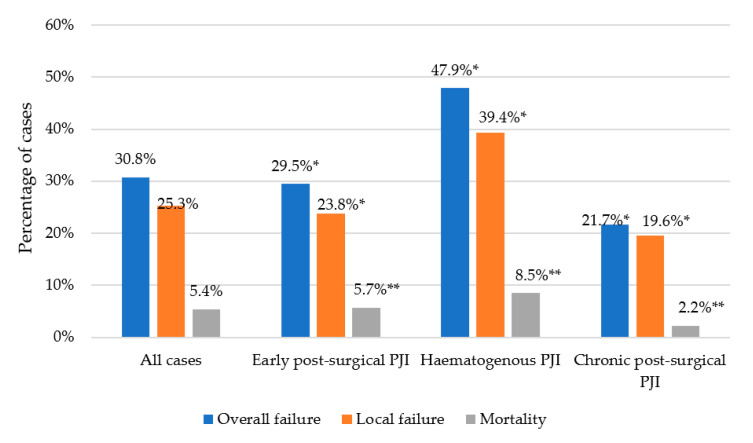
Outcomes of cases with prosthetic joint infection (PJI) by *Staphylococcus aureus* according to the type of infection. * *p* value < 0.01 in overall and local failure between hematogenous, early post-surgical, and chronic post-surgical PJI; ** *p* value = 0.201 in mortality between hematogenous, early post-surgical, and chronic post-surgical PJI.

Follow-up data (median 781 days, interquartile range [IQR] 355-1375) and/or known outcomes were available for 478 cases. Figure 1 shows the percentage of cases with overall failure (local failure plus mortality), local failure, and mortality in all cases and according to the type of PJI.

### 2.1. Implant Removal as the Initial Surgical Strategy in the Cohort of Acute Prosthetic Joint Infection (PJI)

Similar differences in characteristics between cases managed with IRm and DAIR were found in EA and hematogenous PJI, which were therefore analyzed together as acute PJI (*n* = 400, Table 1). Cases with acute PJI managed with IRm were more likely to have a hemiarthroplasty or hematogenous PJI, but also other factors such as abnormal radiography, symptoms duration >21 days, poor condition of soft tissues, or infection by MRSA.

Mortality was greater in acute PJI managed initially with IRm compared to DAIR. However, after performing propensity score matching including several pre-surgical variables (age, number of comorbidities, hemiarthroplasty, hematogenous PJI, abnormal radiography, symptoms duration, condition of soft tissues, infection by MRSA and hospital), mortality was not associated with the IRm procedure itself (OR 1.55; 95%CI 0.47–4.56; *p* = 0.387) (Figure S1A). 

**Figure 2:**
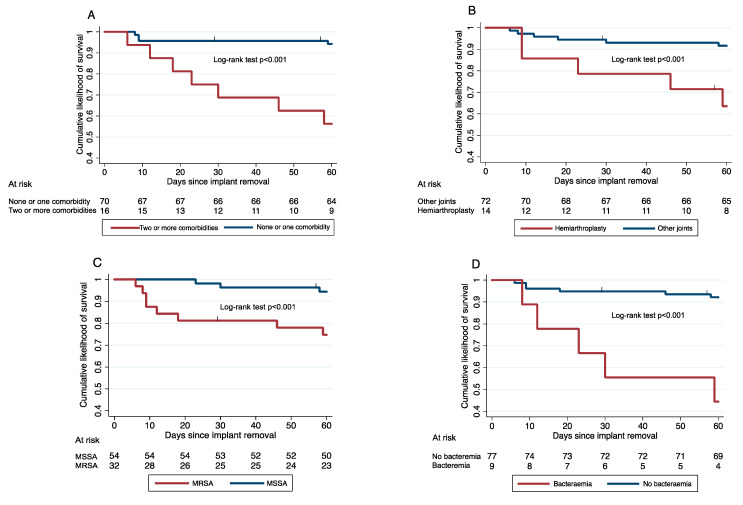
Kaplan–Meier curves of mortality of cases with acute prosthetic joint infection by *Staphylococcus aureus* managed initially with implant removal. (**A**) Patients with and without two or more comorbidities. (**B**) Patients with and without hemiarthroplasty. (**C**) Patients with infection by methicillin-resistant (MRSA) or methicillin-susceptible *S. aureus* (MSSA). (**D**) Patients with and without bacteremia.

Among cases with acute PJI initially managed with IRm (Figure 2), mortality was greater if they had two or more comorbidities (7/16 [43.8%] vs. 4/70 [5.7%]; *p* < 0.001), especially rheumatoid arthritis (3/7 [42.9%] vs. 8/79 [10.1%]; *p* = 0.042) and immunosuppressive treatment (4/7 [57.1%] vs. 7/79 [8.9%]; *p* = 0.004). Mortality was also higher if they had a hemiarthroplasty (5/14 [35.7%] vs. 6/72 [8.3%]; *p* = 0.015), bacteremia (5/9 [55.6%] vs 6/77 [7.8%]; *p* = 0.001), and infection by MRSA (8/32 [25.0%] vs. 3/54 [5.6%]; *p* = 0.016).

### 2.2. Cohort of All Cases Managed with Implant Removal (Initially or Salvage Therapy)

Together with 161 cases managed initially with IRm (63, 26, and 72 with EA, hematogenous and chronic post-surgical PJI, respectively), there were 88 cases (78 with acute and 10 with chronic post-surgical PJI) who finally underwent IRm as salvage therapy. Thus, this procedure was performed in 249 cases (Table 2): two-stage exchange (188, 75.5%), hip resection arthroplasty (44, 17.7%), and one-stage exchange (17, 6.8%). No significant differences were found in surgical strategies between clinical groups (*p* = 0.440). There were 52 cases (27.7%) under the two-stage scheme without a second stage performed; thus, 96 cases (38.6%) finally had resection arthroplasty, who more often had two or more comorbidities (26.0% vs. 16.3%; *p* = 0.063). 

The median length of antimicrobial therapy was 59 days (IQR 43–92). There were 119 cases (55.4%) who received rifampin in combination during ≥21 days in the first 42 days after IRm. Other antibiotics commonly given, either alone or in combination with rifampin, were quinolones (43.3%), beta-lactams (28.8%), cotrimoxazole (16.3%), and glycopeptides (11.6%). 

Overall, 237 cases had outcome data, of whom 37 (15.6%; 95%CI 11.2–20.9) presented overall failure, 22 (9.3%; 95%CI 5.9–13.7) local failure, and 15 patients died (6.3%; 95%CI 3.6–10.2). Mortality occurred to 11 patients (12.8%) with acute PJI initially managed with IRm, two (2.7%) with acute PJI requiring IRm as salvage therapy and two (3.0%) with chronic post-surgical PJI initially managed with IRm. 

Local failure was similar in all IRm strategies, but slightly higher (22.2%) in those with chronic PJI initially managed with DAIR (Table 2). In an analysis of predictive factors of local failure (Table 3), having two or more comorbidities showed a trend toward greater local failure, whereas cases requiring IRm as salvage therapy after DAIR failure did not present worse outcomes. Cases receiving rifampin for 21 days or longer within the first 42 days did not present lower rates of local failure (10.1% vs. 7.3%; *p* = 0.473). Similar results (HR 0.82; 95%CI 0.39–1.70; *p* = 0.590) were found when estimating the effect of rifampin ≥ 21 days after propensity score matching (including age, number of comorbidities, liver cirrhosis, type of infection, infection by MRSA, previous DAIR, type of IRm, and hospital; Figure S1B).

Among the cases with local failure, nine presented symptomatic persistence of infection, eight relapsed, and five presented positive cultures in a second-stage exchange. There were 16 cases with positive staphylococcal cultures upon failure, but none were rifampin-resistant. Long-term follow-up was available in 19/22 cases; four needed long-term SAT, while the rest eventually were considered cured after further treatment. 

## 3. Discussion

PJI by *S. aureus* represents a therapeutic challenge for physicians. While most of the knowledge on its outcome involves patients managed with DAIR [5,6,7,8,9], IRm has received scarce attention in the literature [10,11]. To the best of our knowledge, the present study includes the largest series of cases with *S. aureus* PJI managed with IRm.

The selection of patients with acute PJI (early post-surgical or hematogenous PJI) to be managed either with DAIR or IRm usually follows well-known algorithms such as the standardized Zimmerli criteria [3], which do not include host conditions but factors related to symptom duration, the condition of the implant and soft tissues, and anti-biofilm antimicrobial susceptibility. Additionally, there is still controversy whether DAIR should be performed in infections within 30–90 days since arthroplasty. In this study, among cases with acute PJI, those with hemiarthroplasty, hematogenous PJI, and/or infection by MRSA were more likely to be managed with IRm. 

In this line, previous studies found higher failure rates in patients managed with DAIR who presented these characteristics [8,16] as well as those with particular comorbidities, suggesting that Zimmerli criteria may be revisited. Some authors have attempted to build scores such as the KLIC score [16,17], which may provide guidance in selecting the optimal surgical management for patients with acute PJI. Similarly, the McPherson staging system [15], which includes host factors, has been correlated with the outcome of acute and chronic PJI [18,19] and may define the optimal surgical strategy for each patient [20]. However, these scores were built from studies that include heterogeneous patients with diverse causative microorganisms and have shown poor prediction in staphylococcal PJI. Overall, it seems plausible that future research should address the development of specific scores for *S. aureus* PJI. Additionally, the surgeon’s and/or patient’s preferences should also be considered.

Mortality was higher in patients with acute PJI managed with IRm compared to those undergoing DAIR. Importantly, the surgical procedure was not associated with the higher mortality observed after propensity score matching. Interestingly, the same characteristics (hemiarthroplasty, MRSA) that were driving IRm in acute PJI were also associated with a greater probability of mortality. While some characteristics such as hemiarthroplasty and/or MRSA have been previously recognized as risk factors for mortality [21,22], the results suggest that patients with several underlying conditions also have greater likelihood of mortality, especially if bacteremic [23]. Importantly, these factors may present together [22] and, therefore, physicians should rapidly identify and provide accurate care of older patients, with hemiarthroplasties and/or infections by MRSA when managing acute staphylococcal PJI. 

IRm was associated with low local failure. Obviously, the physical removal of biofilm facilitates the activity of antimicrobials, resulting in a greater chance of cure, compared to DAIR [24,25]. However, even in this favorable situation, some patients failed. Salvage therapy eventually cured most patients, suggesting a good overall prognosis when a first procedure is unsuccessful. 

Most factors associated with mortality did not influence the likelihood of local failure, but cases with several comorbidities had slightly higher local failure [15]. Interestingly, the outcome was not worse in patients who needed IRm as salvage therapy, suggesting that DAIR can be attempted without affecting the prospects for a future removal surgery, if needed. This study, though, could not evaluate whether an initial DAIR might affect the functional outcome of patients needing IRm, which has aroused some controversy in the literature [12]. Failure rates were similar according to IRm strategies including one-stage exchange, as reported also by Senneville et al. [11]. However, since the vast majority of our patients were managed with two-stage exchange, more data are needed to evaluate the outcome of other strategies with larger sample sizes. 

The role of rifampin following IRm is not well established, in contrast with staphylococcal PJI managed with DAIR. In a short series evaluating cases managed with DAIR or IRm [11], Senneville et al. reported better results in patients receiving rifampin, but unfortunately, the authors did not provide a thorough analysis of cases managed with IRm. In this study, a better outcome in patients treated with rifampin during more than 21 days could not be proven. The design of this study did not allow us to draw definitive conclusions on the benefits of rifampin in this setting and further research should address this clinical question.

Several limitations are inherent to the observational retrospective study design, despite being multicentric and its large sample size. Patients included were potentially heterogeneous in their characteristics, presentations, and management, which may have underpowered some analyses. Matching and multivariate analyses have been performed to adjust for this variability, but possible biases and imbalances may still have occurred. Local failure was evaluated based solely on persistence/relapse of *S. aureus*; thus, higher failure rates may have been found if other criteria such as superinfections or orthopedic problems had been included. Finally, not only monomicrobial PJIs by *S. aureus* were included, but also polymicrobial infections. However, we believe that the present data offer an overall perspective of the prognosis of PJI by *S. aureus*.

## 4. Materials and Methods

### 4.1. Design, Setting, and Patients

This was a retrospective, multicenter cohort study performed in 17 hospitals in Spain, in the framework of the Spanish Network for Research in Infectious Diseases (REIPI) during 2003–2010, which included all consecutive cases of PJI caused by *S. aureus* identified from previously registered databases or from the general archives in each hospital. Two cohorts were analyzed: (i) the subcohort of acute PJI was used to compare the characteristics and outcomes of cases managed initially with IRm or DAIR; and (ii) the subcohort of all cases managed with IRm, either initially or as salvage therapy after DAIR failure, was used to investigate their outcome (mortality and factors predicting failure).

Cases of PJI caused by *S. aureus*, monomicrobial or polymicrobial, managed with DAIR or IRm were included. Cases where *S. aureus* did not cause the original PJI, but participated later as a superinfecting microorganism, those requiring amputation as the initial surgical procedure for IRm, and those catalogued as positive intraoperative cultures according to Tsukayama’s criteria [26] were excluded. Patient consent was not required, given the retrospective design; data were anonymized, without sensitive information that may enable the participant’s identification. 

### 4.2. Definitions 

PJI was defined according to Infectious Diseases Society of America guidelines and microorganisms were identified according to standard criteria [3,4,8,27]. In accordance with the most commonly used classifications of types of PJI, these were categorized into early post-surgical (EA), chronic post-surgical, and acute hematogenous. The latter included cases with ≤3 weeks of symptoms duration appearing three months after surgery in the setting of microbiologically confirmed or clinically suspected staphylococcal bacteremia. Regarding EA and chronic post-surgical PJI, there is still controversy on definitions based on time from arthroplasty and accordingly, EA-PJI may include cases that present within one month or three months [3,4,26,28,29], and these time cut-offs are usually employed in selecting patients to be managed successfully with DAIR. Thus, for the purpose of this study, PJI occurring within three months after prosthesis placement were classified as EA, whereas defined as chronic post-surgical, if these started thereafter [3,4]. When analyzing the subcohort of acute PJI, EA and acute hematogenous PJI were included. 

Baseline characteristics were recorded and included severe comorbidities (diabetes, liver cirrhosis, chronic kidney disease (CKD), immunosuppressive treatment, rheumatoid arthritis, malignancy, chronic lung, and heart diseases) present in the McPherson Staging System [15]. 

### 4.3. Clinical and Surgical Management

The decision to manage patients with DAIR or IRm was taken by the attending medical team, commonly following Zimmerli’s criteria [3]; patients with duration of symptoms ≤21 days, a stable implant, and appropriate soft tissues condition usually qualify for DAIR. Regarding the controversies in definitions of EA PJI above-mentioned, infections occurring within 30–90 days since prosthesis placement were usually considered for IRm, but might have also been managed with DAIR. 

DAIR management, which was performed only as an initial strategy, has been described elsewhere [7,8]. IRm was performed as an initial strategy or after DAIR in patients who failed. IRm was classified into three surgical approaches [30]: (a) two-stage exchange; (b) one-stage exchange; and (c) hip resection arthroplasty. Cases were considered under the two-stage exchange scheme if the intention was to implant a new prosthesis or arthrodesis, irrespective of whether this second stage was finally performed.

In most hospitals, the usual perioperative antimicrobial prophylaxis for arthroplasties consists of intravenous cefazolin 2 g. After the surgical procedure for PJI, intravenous antibiotics of wide antimicrobial-spectrum are administered. Once the antimicrobial susceptibility is available, antibiotics are adjusted according to current guidelines. However, the ultimate choice of the antimicrobial treatment is at the discretion of the medical team. The intravenous route is maintained for a variable period depending on each hospital protocol, usually followed by oral antibiotics, also for a variable time.

### 4.4. Outcomes and Follow-Up

Patients were followed until death, failure, or loss to follow-up. Overall failure was defined as a composite endpoint consisting of local failure and/or mortality due to any cause occurring within 60 days since surgery (cut-off selected to reflect mortality potentially linked to the PJI process). 

In cases managed with DAIR, local failure has been defined elsewhere [8], but only considered if related to staphylococcal persistence/relapse. In cases managed with IRm, it was defined also only if staphylococcal persistence/relapse as: (a) symptom persistence beginning within 30 days after IRm, leading to long-term suppressive antimicrobial therapy (SAT) and/or new surgeries, irrespective of when these were performed; (b) relapsing symptoms in asymptomatic patients initially considered cured after IRm; and (c) positive *S. aureus* cultures in asymptomatic patients undergoing a second-stage surgery. 

### 4.5. Statistical Analysis

Data were analyzed with Stata 13.1 (Stata Corporation, USA). Categorical and continuous variables were described by counts and percentages, and median and interquartile range (IQR), respectively. Comparisons between groups were performed with the chi-square test or Fisher exact test for categorical variables and the t-test or Mann–Whitney test for continuous variables.

Multivariate logistic regression was used to analyze factors associated with initial management with IRm in acute PJI including the commonly used Zimmerli’s criteria [3]. Kaplan–Meier survival curves were used to evaluate the probability of success during follow-up and the log-rank test analyzed differences between groups, censoring cases lost to follow-up. Multivariate Cox regression was performed to estimate factors associated with local failure, censoring death as a competing event. 

To evaluate the impact of interventions (surgical procedure [DAIR vs. IRm] and rifampin) on mortality and local failure, respectively, propensity score matching analyses were performed. Clinically relevant variables were introduced in the propensity model, together with baseline characteristics found to have a univariate association with the intervention (*p* < 0.1). The adequacy of the models was assessed with calibration plots and the Hosmer–Lemeshow test. Nearest neighbor matching with replacement was performed with 0.1 calipers. Mean standardized differences for covariates between matched groups were checked prior to treatment effects estimation.

The length of antibiotic therapy could be shortened in cases failing prematurely and would not actually be the cause of failure but its consequence. Thus, in order to avoid survivor’s bias, the influence of rifampin on local failure was only analyzed in cases treated for ≥21 days and not requiring salvage surgeries within the first 42 days after IRm.

## 5. Conclusions

In conclusion, while mortality was significant in acute PJI by *S. aureus* managed with IRm, there was no evident association with the surgical approach itself. Additionally, we identified factors related to the patient’s condition that were associated with a greater probability of death among these cases. Local failure was low, but a previous DAIR strategy did not worsen the outcome of cases. Despite the limited efficacy found in this study, further research should confirm whether rifampin may still offer a potential benefit in the treatment of patients with staphylococcal PJI managed with IRm.

## Figures and Tables

**Table 1:** Characteristics, adjusted odds ratios of implant removal and outcome of 400 cases of acute prosthetic joint infection (early post-surgical and hematogenous) by *Staphylococcus aureus*, according to their initial surgical management.

Characteristic	Acute PJI Managed with DAIR (*n* = 311)	Acute PJI Managed with Implant Removal (*n* = 89)	*p* Value	Adjusted OR of Implant Removal (95% CI) *	*p* Value
PATIENT CHARACTERISTICS					
Female sex	184 (59.2)	59 (66.3)	0.225		
Age (years) ^1^	72 (64–78)	74 (68–78)	0.168	1.01 (0.99–1.04)	0.294
Two or more comorbidities ^2^	60 (19.3)	17 (19.1)	0.968		
Chronic kidney disease	16 (5.1)	8 (9.0)	0.178		
PROSTHESIS CHARACTERISTICS					
Total hip arthroplasty	109 (35.1)	27 (30.3)	0.408		
Total knee arthroplasty	174 (56.0)	45 (50.6)	0.368		
Hemiarthroplasty	24 (7.7)	14 (15.7)	0.023	3.73 (1.60–8.68)	0.003
Revision prosthesis	53 (17.0)	14 (15.7)	0.770		
CLINICAL AND ANALYTICAL DATA					
Hematogenous PJI	49 (15.8)	26 (29.2)	0.004	3.34 (1.72–6.46)	<0.001
Leukocytosis ^3^	157 (50.5)	52 (58.4)	0.186		
CRP (mg/L)	109 (28–120)	100 (30–107)	0.415		
Abnormal radiography ^4^	27 (8.7)	26 (29.2)	<0.001	3.70 (1.85–7.43)	<0.001
Duration of symptoms > 21 days	33 (10.6)	37 (41.6)	<0.001	7.69 (4.03–14.68)	<0.001
Poor condition of soft tissues	37 (11.9)	19 (21.4)	0.023	1.50 (0.72–3.12)	0.282
Infection by MRSA	75 (24.1)	34 (38.2)	0.008	1.42 (0.95–3.19)	0.074
Bacteremia	57 (18.3)	17 (19.1)	0.868		
Polymicrobial infection	58 (18.7)	19 (21.4)	0.569		
OUTCOME ^5^					
Overall failure	107 (35.7)	20 (23.3)	0.031		
Local failure	94 (31.3)	9 (10.5)	<0.001		
Mortality <60 days	13 (4.3)	11 (12.8)	0.004		

Categorical variables expressed in absolute number and (percentage); continuous variables expressed in median and (interquartile range). PJI: Prosthetic joint infection. DAIR: Debridement, antibiotics and implant retention. OR: Odds ratio. 95%CI: 95% Confidence Interval. IQR: Interquartile range. MRSA: Methicillin-resistant *S. aureus*. * Refers to a multivariate analysis of factors associated with implant removal in patients with acute PJI (*n* = 400). ^1^ Odds ratio expressed as per year. ^2^ These include severe comorbidities (diabetes, liver cirrhosis, chronic kidney disease, immunosuppressive treatment, rheumatoid arthritis, malignancy, chronic lung, and heart diseases) present in the McPherson Staging System [15]. ^3^ Leukocytosis is defined as baseline leukocyte count above 10 × 10^9^/L. ^4^ Abnormal radiography is defined according to radiologic signs of infection (loosening, periprosthetic osteolysis, migration, subperiostic reaction). ^5^ Calculated among 300 patients with acute PJI managed with DAIR and 86 patients with acute PJI managed with implant removal.

**Table 2:** Characteristics and outcome of the subcohort of cases of prosthetic joint infection by *Staphylococcus aureus* managed with implant removal (*n* = 249), according to their type of infection.

	Acute PJI (Early Post-Surgical and Hematogenous)			Chronic Post-Surgical PJI		
Characteristic	IRm as Initial Strategy (*n* = 89)	IRm as Salvage Therapy (*n* = 78)	*p* Value *	IRm as Initial Strategy (*n* = 72)	IRm as Salvage Therapy (*n* = 10)	*p* Value **
Female sex	59 (66.3)	52 (66.7)	0.959	46 (63.9)	7 (70.0)	0.705
Age (years)	74 (68–78)	72 (60–79)	0.470	74 (64–79)	68 (57–76)	0.082
Two or more comorbidities ^1^	17 (19.1)	16 (20.5)	0.819	13 (18.1)	4 (40.0)	0.109
Chronic kidney disease	8 (9.0)	6 (7.7)	0.763	8 (11.1)	0	0.267
Hemiarthroplasty	14 (15.7)	3 (3.9)	0.011	3 (4.2)	0	0.511
Revision prosthesis	14 (15.7)	18 (23.1)	0.229	15 (20.8)	5 (50.0)	0.044
Infection by MRSA	34 (38.2)	16 (20.5)	0.013	12 (16.7)	3 (30.0)	0.307
Bacteremia	9 (10.1)	4 (5.1)	0.230	1 (1.4)	0	0.708
Polymicrobial infection	19 (21.4)	20 (25.6)	0.513	20 (27.8)	2 (20.0)	0.603
OUTCOME ^2^						
Overall failure	20 (23.3)	9 (12.0)	0.064	6 (9.0)	2 (22.2)	0.223
Local failure	9 (10.5)	7 (9.3)	0.811	4 (6.0)	2 (22.2)	0.090
Mortality < 60 days	11 (12.8)	2 (2.7)	0.019	2 (3.0)	0	0.599

Categorical variables expressed in absolute number and (percentage); continuous variables expressed in median and (interquartile range). IRm as salvage therapy refer to cases who failed after an initial management with DAIR (Debridement, antibiotics, and implant retention). PJI: Prosthetic joint infection. IRm: Implant removal. MRSA: Methicillin-resistant *S. aureus*. * Comparison between acute PJI. ** Comparison between chronic PJI. ^1^ These include severe comorbidities (diabetes, liver cirrhosis, chronic kidney disease, immunosuppressive treatment, rheumatoid arthritis, malignancy, chronic lung and heart diseases) present in the McPherson Staging System [15]. ^2^ Calculated among 86 patients with acute PJI initially managed with implant removal, 75 patients with acute PJI initially managed with DAIR, 67 patients with chronic PJI initially managed with implant removal, and nine patients with chronic PJI initially managed with DAIR.

**Table 3:** Predictive factors of local failure among 237 cases of prosthetic joint infection by *Staphylococcus aureus* managed with implant removal (22 cases failed).

Characteristic		Failed/Total (%)	Crude HR (95%CI)	*p* Value	Adjusted HR (95% CI)	*p* Value
Age	<75 years	15/127 (11.8)	1		1	
	≥75 years	7/110 (6.4)	0.59 (0.24–1.44)	0.234	0.59 (0.24–1.44)	0.231
Sex	Male	9/80 (11.3)	1			
	Female	13/157 (8.3)	0.67 (0.29–1.57)	0.364		
Two or more comorbidities ^1^	No	15/192 (7.8)	1		1	
	Yes	7/45 (15.6)	2.44 (0.99–5.99)	0.051	2.46 (1.00–6.09)	0.051
Hemiarthroplasty	No	21/218 (9.6)	1			
	Yes	1/19 (5.3)	0.87 (0.12–6.50)	0.891		
Revision prosthesis	No	15/185 (8.1)	1			
	Yes	7/52 (13.5)	1.77 (0.72–4.37)	0.232		
Hematogenous PJI	No	18/193 (9.3)	1			
	Yes	4/44 (9.1)	0.93 (0.31–2.75)	0.891		
Infection by MRSA	No	18/179 (10.1)	1			
	Yes	4/58 (6.9)	0.87 (0.29–2.57)	0.798		
Bacteremia	No	21/223 (9.4)	1			
	Yes	1/14 (7.1)	1.76 (0.23–13.32)	0.613		
Polymicrobial infection	No	19/178 (10.7)	1			
	Yes	3/59 (5.1)	0.51 (0.15–1.71)	0.236		
Initially managed with DAIR	No	13/153 (8.5)	1		1	
	Yes	9/84 (10.7)	1.06 (0.45–2.49)	0.886	0.94 (0.40–2.23)	0.897
Surgical management	One-stage exchange	2/16 (12.5)	1			
	Two-stage exchange	16/182 (8.8)	0.60 (0.14–2.62)			
	Hip resection arthroplasty	4/39 (10.3)	0.84 (0.15–4.61)	0.720		
Rifampin ^2^	No	7/96 (7.3)	1			
	Yes	12/119 (10.1)	1.01 (0.37–2.73)	0.989		

HR: Hazard ratio. 95% CI: 95% Confidence interval. PJI: Prosthetic joint infection. MRSA: Methicillin-resistant *S. aureus*. DAIR: Debridement, antibiotics, and implant retention. ^1^ These include severe comorbidities (diabetes, liver cirrhosis, chronic kidney disease, immunosuppressive treatment, rheumatoid arthritis, malignancy, chronic lung and heart diseases) present in the McPherson Staging System [15]. ^2^ Among those treated with rifampin for 21 days or longer in the initial 42 days since implant removal.

## Data Availability

The data presented in this study are available upon request from the corresponding author.

## Supplementary Materials

The following are available online at https://www.mdpi.com/2079-6382/10/2/118/s1, Figure S1: Standardized bias for covariates before and after propensity score matching for the evaluation of implant removal on mortality in cases with acute prosthetic joint infection by *Staphylococcus aureus* (A) and the role of rifampin on local failure in all cases managed with implant removal (B).

## References

[1] Del Pozo J.L., Patel R. (2009). Clinical practice. Infection associated with prosthetic joints. N. Engl. J. Med..

[2] Benito N., Franco M., Ribera A., Soriano A., Rodriguez-Pardo D., Sorli L., Fresco G., Fernandez-Sampedro M., Dolores Del Toro M., Guio L. (2016). Time trends in the aetiology of prosthetic joint infections: A multicentre cohort study. Clin. Microbiol. Infect..

[3] Zimmerli W., Trampuz A., Ochsner P.E. (2004). Prosthetic-joint infections. N. Engl. J. Med..

[4] Osmon D.R., Berbari E.F., Berendt A.R., Lew D., Zimmerli W., Steckelberg J.M., Rao N., Hanssen A., Wilson W.R. (2013). Diagnosis and management of prosthetic joint infection: Clinical practice guidelines by the Infectious Diseases Society of America. Clin. Infect. Dis..

[5] Aboltins C.A., Page M.A., Buising K.L., Jenney A.W., Daffy J.R., Choong P.F., Stanley P.A. (2007). Treatment of staphylococcal prosthetic joint infections with debridement, prosthesis retention and oral rifampicin and fusidic acid. Clin. Microbiol. Infect..

[6] Brandt C.M., Sistrunk W.W., Duffy M.C., Hanssen A.D., Steckelberg J.M., Ilstrup D.M., Osmon D.R. (1997). Staphylococcus aureus prosthetic joint infection treated with debridement and prosthesis retention. Clin. Infect. Dis..

[7] Byren I., Bejon P., Atkins B.L., Angus B., Masters S., McLardy-Smith P., Gundle R., Berendt A. (2009). One hundred and twelve infected arthroplasties treated with ‘DAIR’ (debridement, antibiotics and implant retention): Antibiotic duration and outcome. J. Antimicrob. Chemother..

[8] Lora-Tamayo J., Murillo O., Iribarren J.A., Soriano A., Sanchez-Somolinos M., Baraia-Etxaburu J.M., Rico A., Palomino J., Rodriguez-Pardo D., Horcajada J.P. (2013). A large multicenter study of methicillin-susceptible and methicillin-resistant Staphylococcus aureus prosthetic joint infections managed with implant retention. Clin. Infect. Dis..

[9] Marculescu C.E., Berbari E.F., Hanssen A.D., Steckelberg J.M., Harmsen S.W., Mandrekar J.N., Osmon D.R. (2006). Outcome of prosthetic joint infections treated with debridement and retention of components. Clin. Infect. Dis..

[10] Brandt C.M., Duffy M.C., Berbari E.F., Hanssen A.D., Steckelberg J.M., Osmon D.R. (1999). Staphylococcus aureus prosthetic joint infection treated with prosthesis removal and delayed reimplantation arthroplasty. Mayo. Clin. Proc..

[11] Senneville E., Joulie D., Legout L., Valette M., Dezeque H., Beltrand E., Rosele B., d’Escrivan T., Loiez C., Caillaux M. (2011). Outcome and predictors of treatment failure in total hip/knee prosthetic joint infections due to Staphylococcus aureus. Clin. Infect. Dis..

[12] Rajgopal A., Panda I., Rao A., Dahiya V., Gupta H. (2018). Does Prior Failed Debridement Compromise the Outcome of Subsequent Two- Stage Revision Done for Periprosthetic Joint Infection Following Total Knee Arthroplasty?. J. Arthroplast..

[13] Sherrell J.C., Fehring T.K., Odum S., Hansen E., Zmistowski B., Dennos A., Kalore N. (2011). The Chitranjan Ranawat Award: Fate of two-stage reimplantation after failed irrigation and debridement for periprosthetic knee infection. Clin. Orthop. Relat. Res..

[14] Zimmerli W., Widmer A.F., Blatter M., Frei R., Ochsner P.E. (1998). Role of rifampin for treatment of orthopedic implant-related staphylococcal infections: A randomized controlled trial. Foreign-Body Infection (FBI) Study Group. Jama.

[15] McPherson E.J., Woodson C., Holtom P., Roidis N., Shufelt C., Patzakis M. (2002). Periprosthetic total hip infection: Outcomes using a staging system. Clin. Orthop. Relat. Res..

[16] Wouthuyzen-Bakker M., Sebillotte M., Lomas J., Taylor A., Palomares E.B., Murillo O., Parvizi J., Shohat N., Reinoso J.C., Sanchez R.E. (2019). Clinical outcome and risk factors for failure in late acute prosthetic joint infections treated with debridement and implant retention. J. Infect..

[17] Tornero E., Morata L., Martinez-Pastor J.C., Bori G., Climent C., Garcia-Velez D.M., Garcia-Ramiro S., Bosch J., Mensa J., Soriano A. (2015). KLIC-score for predicting early failure in prosthetic joint infections treated with debridement, implant retention and antibiotics. Clin. Microbiol. Infect..

[18] Bryan A.J., Abdel M.P., Sanders T.L., Fitzgerald S.F., Hanssen A.D., Berry D.J. (2017). Irrigation and Debridement with Component Retention for Acute Infection After Hip Arthroplasty: Improved Results with Contemporary Management. J. Bone Joint Surg. Am..

[19] Wimmer M.D., Randau T.M., Friedrich M.J., Ploeger M.M., Schmolder J., Strauss A.C., Pennekamp P.H., Vavken P., Gravius S. (2016). Outcome Predictors in Prosthetic Joint Infections--Validation of a risk stratification score for Prosthetic Joint Infections in 120 cases. Acta Orthop. Belg..

[20] Amanatullah D., Dennis D., Oltra E.G., Marcelino Gomes L.S., Goodman S.B., Hamlin B., Hansen E., Hashemi-Nejad A., Holst D.C., Komnos G. (2019). Hip and Knee Section, Diagnosis, Definitions: Proceedings of International Consensus on Orthopedic Infections. J. Arthroplast..

[21] Gomez-Junyent J., Murillo O., Grau I., Benavent E., Ribera A., Cabo X., Tubau F., Ariza J., Pallares R. (2018). Analysis of mortality in a cohort of 650 cases of bacteremic osteoarticular infections. Semin. Arthritis Rheum..

[22] Lora-Tamayo J., Euba G., Ribera A., Murillo O., Pedrero S., Garcia-Somoza D., Pujol M., Cabo X., Ariza J. (2013). Infected hip hemiarthroplasties and total hip arthroplasties: Differential findings and prognosis. J. Infect..

[23] Kurtz S.M., Lau E.C., Son M.S., Chang E.T., Zimmerli W., Parvizi J. (2018). Are We Winning or Losing the Battle With Periprosthetic Joint Infection: Trends in Periprosthetic Joint Infection and Mortality Risk for the Medicare Population. J. Arthroplast..

[24] Hart W.J., Jones R.S. (2006). Two-stage revision of infected total knee replacements using articulating cement spacers and short-term antibiotic therapy. J. Bone Joint Surg. Br..

[25] Tibrewal S., Malagelada F., Jeyaseelan L., Posch F., Scott G. (2014). Single-stage revision for the infected total knee replacement: Results from a single centre. Bone Joint J..

[26] Tsukayama D.T., Estrada R., Gustilo R.B. (1996). Infection after total hip arthroplasty. A study of the treatment of one hundred and six infections. J. Bone Joint Surg. Am..

[27] Murray P.R., Jo Baron E., Jorgensen J.H., Landry M.I., Pfaller M.A. (2007). Manual of Clinical Microbiology.

[28] Chotanaphuti T., Courtney P.M., Fram B., In den Kleef N.J., Kim T.K., Kuo F.C., Lustig S., Moojen D.J., Nijhof M., Oliashirazi A. (2019). Hip and Knee Section, Treatment, Algorithm: Proceedings of International Consensus on Orthopedic Infections. J. Arthroplast..

[29] Zimmerli W., Sendi P. (2015). Orthopedic Implant-Associated Infections. Mandell, Douglas, and Bennett’s Principles and Practice of Infectious Diseases.

[30] Tande A.J., Patel R. (2014). Prosthetic joint infection. Clin. Microbiol. Rev..

