# Supplementary material for: Implant Removal in the Management of Prosthetic Joint Infection by Staphylococcus aureus: Outcome and Predictors of Failure in a Large Retrospective Multicenter Study
Source: Antibiotics (Basel). 2021 Jan 26;10(2):118. doi: 10.3390/antibiotics10020118 (PMC7911003; doi:10.3390/antibiotics10020118)
Supplement: Supplementary file 1 [file antibiotics-10-00118-s001.pdf]

antibiotics

MDPI

Figure 1. Standardized bias for covariates before and after propensity score matching for the evaluation of implant removal on mortality in cases with acute prosthetic joint infection by *Staphylococcus aureus* (A) and the role of rifampin on local failure in all cases managed with implant removal (B).

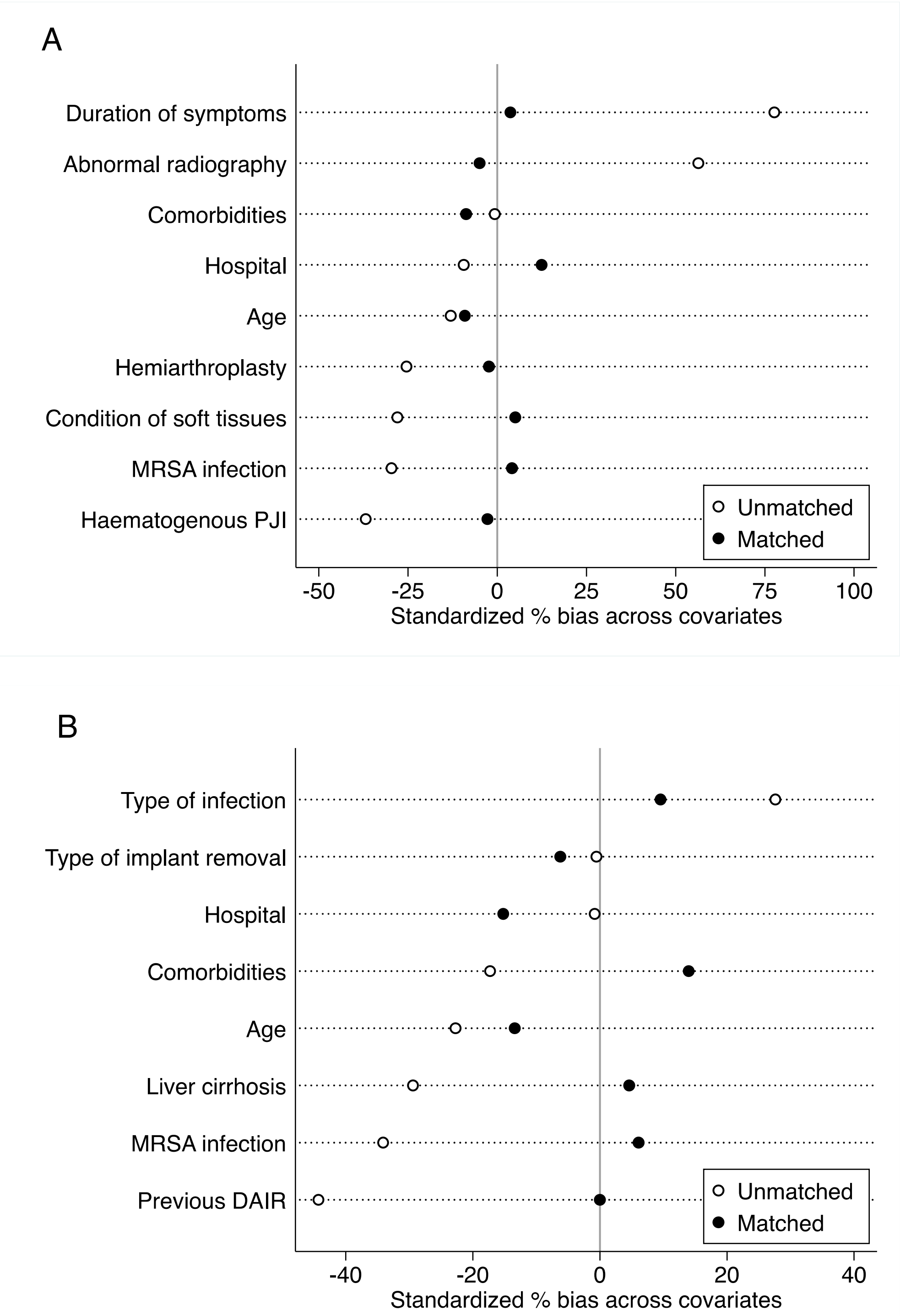

Abbreviations: MRSA: Methicillin-resistant *S. aureus*; DAIR: Debridement, antibiotics and implant retention.
